# Comparative evaluation and patient satisfaction with an electrical impedance-based device versus digital radiography in the estimation of remaining dentin thickness in carious posterior permanent teeth: (Diagnostic accuracy study)

**DOI:** 10.1186/s12903-024-04205-w

**Published:** 2024-04-08

**Authors:** Rawda H. Abd ElAziz, Rasha A. Ragab, Ghada A. Elzayat

**Affiliations:** 1 Conservative Dentistry Department, Faculty of Dentistry, Cairo University, 11 EL-Saraya St. Elmanial, Cairo, Postal Code: 11553 Egypt; 2 Pediatric Dentistry Department, Faculty of Dentistry, Cairo University, 11 EL-Saraya St. Elmanial, Cairo, Postal Code: 11553 Egypt; 3 Conservative Dentistry Department, Faculty of Dentistry, Egyptian Russian University, Badr City, Cairo, Postal Code: 11829 Egypt

**Keywords:** Dental caries, Electric impedance, Dentin electric resistance based tool, Periapical digital radiography, Patient-reported outcome measures, Diagnostic accuracy study, Remaining dentin thickness (RDT), Patient satisfaction

## Abstract

**Background:**

Accurate assessment of remaining dentin thickness (RDT) is paramount for restorative decisions and treatment planning of vital teeth to avoid any pulpal injury. This diagnostic accuracy study compared the validity and patient satisfaction of an electrical impedance based device Prepometer™ (Hager & Werken, Duisburg, Germany) versus intraoral digital radiography for the estimation of remaining dentin thickness in carious posterior permanent teeth.

**Methods:**

Seventy patients aged 12–25 years with carious occlusal or proximal permanent vital posterior teeth were recruited. Tooth preparation was performed to receive an adhesive restoration. Pre- and post-excavation RDT were measured radiographically by two calibrated raters using the paralleling periapical technique. Prepometer™ measurements were performed by the operator. Patients rated their satisfaction level with each tool on a 4-point Likert scale and 100 mm visual analog scale (VAS). Inter and intragroup comparisons were analyzed using signed rank test, while agreement between devices and observations was tested using weight kappa (WK) coefficient.

**Results:**

the intergroup comparisons showed that, before and after excavation, there was a significant difference between measurements made by both techniques (*p* < 0.001). After excavation, there was a weak agreement between measurements (WK = 0.2, *p* < 0.001), whereas before excavation, the agreement was not statistically significant (*p* = 0.407). Patients were significantly more satisfied with Prepometer™ based on scales and VAS (*p* < 0.001).

**Conclusion:**

Prepometer™ could be a viable clinical tool for determining RDT with high patient satisfaction, while radiographs tended to overestimate RDT in relation to the Prepometer™.

## Introduction

Successful restorative treatment of vital teeth depends on a holistic understanding of the tooth structure, mechanical and esthetic characteristics as well as function of the dentin-pulp complex which forms the necessary biological foundation for any restorative decision [1]. Since the best protective barrier for the pulp against trauma is the healthy dentin, it is very crucial during tooth preparation to consider the remaining dentin thickness (RDT). In the literature, an RDT of 2 mm is thought to be perfect for pulp protection [2–4]. Thin RDT less than 1 mm in deep preparations where more and wider dentinal tubules per mm^2^ are exposed, directly increases the risk for pulpal injury, affecting its repair response and subsequent restorative treatment options needed. Whilst in RDT of 0.5 mm, the dentin tubules are numerous, open, and wide enough as if there is a true pulpal exposure, [4]. Thus, it is very crucial to accurately assess the RDT especially in deep caries affected teeth to properly locate the caries excavation endpoint and decide the suitable restorative treatment thus preserving the tooth pulp vitality.

Clinical assessment of RDT is mainly dependent on integrating the operator’s knowledge of tooth anatomy and clinical experience, with radiographic findings interpretation [5]. However, this is very challenging owing to the subjective nature of these parameters. Although the radiographic assessment of RDT is the most available valid tool, it has many limitations such as the inconsistent estimation as Berbari et al., [4] found that it underestimated the real RDT by approximately 20% while on the contrary, Lancaster et al., [6] found that it overestimated the RDT. Besides its hazardous procedures to both the patient and dental personnel, overlapping of the anatomical structures and financial cost. Some other techniques have been investigated to assess RDT like laser fluorescence, pulse-echo, optical coherence tomography (OCT), and cone beam computed tomography (CBCT) however, they are not commonly used in dental practice for such a use [1, 7, 8]. Thus, the need for a real-time monitoring, non-invasive, user friendly and reliable tool is yet the most advocated.

One of the noninvasive clinical methods for RDT determination involves measuring electric resistance of the dentin by devices like Prepometer™ (Hager & Werken, Duisburg, Germany) and EndoEst 3D™ (Geosoft®, Russia), which is a multifunctional tool that combines a dentin meter to measure RDT, an apex locator and a pulp tester [9].

Prepometer™ is introduced to measure RDT only following tooth preparation. It depends on electrical impedance where an alternating 500-Hz electric current with an amplitude of 10A flows between a measuring sensory electrode placed on the prepared dentin and a reference electrode attached to the lip clip. The device also contains a third electrode for calibration. Ten LED lights of three colors like traffic light on the device measure, analyze, and show the resistance within ten seconds for each measurement. The light colors alter as the dentin electrical resistance declines, signaling an elevated danger of pulpal exposure [10].

Many invitro studies have used and evaluated the Prepometer™ [5, 9–12] nevertheless few clinical data are available; [8, 13] thus, this clinical study was introduced to help in proving the validity of this device clinically. Also due to the raised importance of patient-reported outcome measures (PROMs) and lack of sufficient knowledge in this area, patient satisfaction regarding the two devices was measured. The research question was that in patients with carious permanent posterior teeth, would the electrical impedance device be as valid as the digital radiography in clinical estimation of remaining dentin thickness? The proposed hypothesis is null.

## Methods

### Study settings

This clinical trial was conducted in Faculty of Dentistry- Cairo University, Egypt and was implemented following the ethical principles stated in the World Medical Association Declaration of Helsinki. The research protocol was reviewed and approved by the research ethics committee of the faculty with approval number (39–7-2022) on 26/7/2022. It was retrospectively registered on the clinicaltrials.gov website (https://clinicaltrials.gov/) with identification number (NCT06162182) on 8/12/2023. The trial has been reported following the STARD 2015 guidelines for Reporting Diagnostic Accuracy Studies.

### Sample size calculation

A power analysis was designed by adopting an alpha (α) level of 0.05 (5%), a beta (β) level of 0.20 (20%), a within-subject correlation coefficient of (0.4), and a difference in proportions of (0.19) based on the results of a previous study [4] and on expert’s opinion; the predicted sample size (n) was found to be a total of (67) cases.

### Clinical examination

Using a 0.5-mm ball-ended probe (CPITN Probe, Premium Instruments, USA) and a dental mirror, carious lesions were examined and scored according to the International Caries Detection and Assessment System “ICDAS”. Calibration of the examiners was executed using an online program on the International Caries Classification and Management System “ICCMS” website (https://www.iccms-web.com) to accurately define the eligible participants.

### Eligibility criteria

Participants eligible for this study were those aged from 12 to 25 y, willing to join the study and with clinically detectable occlusal or proximal carious ( ICDAS score 3, 4) vital permanent posterior tooth with closed apex and healthy periodontal supporting. The minimum extension of the carious cavity should be at least 1 mm in width to accommodate with the probe of the Prepometer™ [14]. Excluded participants were those with poor oral hygiene, severe medical complications, showing signs and symptoms of irreversible or necrotic pulp pathology or with internal or external root resorption also when the affected tooth was with extended buccal or lingual caries, extending clinically very deep to the inner half of dentin thickness or previously restored. Also, patients suffering from any developmental or formative abnormalities e.g. molar incisor hypomineralization were excluded from the trial.

Patients were informed of the goals and procedures of the trial before consenting to participate and signing the informed consent form.

### Operative procedures

All the operative procedures were performed under local anesthesia (articaine HCL 4% and epinephrine 1:100,000 (Artinibsa; Inibsa, Spain)) by one experienced operator. The tooth was prepared to receive an adhesive restoration. A tungsten carbide bur no. #245 (0.8 mm in diameter and 1.6 mm in length] (Komet, Germany) rotating in a high speed handpiece was used to remove the superficial and undermined enamel to gain access to the carious dentine and to remove caries from the walls for at least the 2 mm of the cavity boundaries to provide the peripheral seal necessary for the restoration success. The clinical determination of the caries removal endpoint was done based on the selective removal of caries consensus [15]. A sharp discoid excavator (#51&52 Dentsply Maillefer, Switzerland) was used to remove remaining carious dentine either to firm dentine “physically resistant to hand excavation and some pressure needs to be exerted through an instrument to lift it” in shallow and moderately deep cavities or to soft dentine “that deforms when an instrument is pressed into it and can be easily scooped up (e.g. with a spoon hand excavator) with little force being required” in deep and very deep cavities [15].

### Measuring the remaining dentin thickness (RDT)

#### Index test: electrical impedance device, Prepometer™ (Hager & Werken, Duisburg, Germany)

**Fig. 1:**
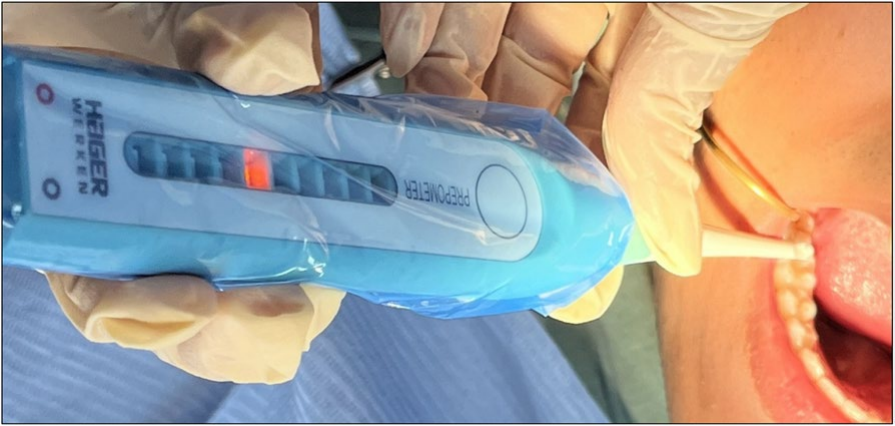
The Prepometer™ measurement of the RDT (Orange LED) for an occlusodistal carious cavity in lower right first molar

The device was calibrated before any measurement by simultaneously touching the wet dentin surface with the calibration and sensor electrodes which is confirmed by the sequential flash of all the LEDs [10]. The reference electrode was placed on the buccal vestibule during the measurement. The sensor electrode was gently dragged across the cavity floor to measure the thickness of the remaining dentin at the deepest area, which was located by two trained raters and pointed by a periodontal probe [8, 14]. The electrical impedance value is displayed on the Prepometer™ by a scale represented by ten LEDs illuminated with different colors. They represent according to the manufacturer: green – a riskless preparation, yellow—further preparation is still possible, orange – limited range for a safe preparation and it should be stopped, finally red – imminent endangerment of pulp vitality [16]. Preoperative and post excavation measurements were taken Fig. 1.

#### Reference standard: intraoral digital radiographic examination

A preoperative digital periapical radiograph using the paralleling technique was taken of the selected tooth using an intraoral X-ray unit (Vario DG, Dentsply Sirona) with an exposure time of 0.88 s, at 70 kV, and a tube current of 3.5 mA using a photostimulable phosphor plate (PSP) film sensor of (31 × 41 mm) dimensions VistaScan® Imaging Plate PLUS, size 2 (Dürr Dental AG, Germany). Another post-excavation periapical radiograph using the same machine and settings was taken upon the completion of caries management. A digital reader preset (VistaScan Mini Plus, Dürr Dental, Germany) was used to process the radiographic images. The image analysis was executed using DBSWIN 5.4.0; a dental imaging processing software and visualized on a LED monitor (Lenovo D19-10 18.5 inch HD, China).

**Fig. 2:**
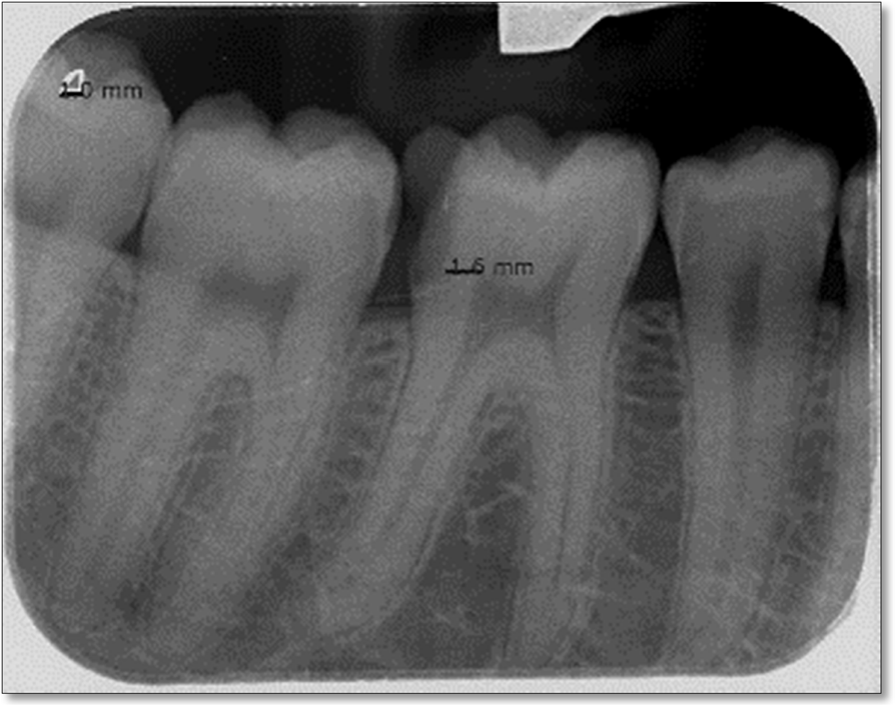
A radiograph showing the measurement of the RDT (1.6 mm) on the distal of the lower right first molar. Note the triangle in the upper border of the radiograph was used as a reference

Two methods of image calibration were employed. First, the program was calibrated by equal matching the length of the imported radiographic photo to the actual length of the film sensor used for the study (41 mm). In addition, a reference guide was used which was the built-in (1 × 1 mm) triangle located at the corner of the used image plate. These calibration procedures allowed for millimeter-scale line measurements of the RDT. Two calibrated raters recorded all the measurements to confirm the reproducibility of the readings and to minimize the possibility of errors. They were blinded to the readings of the electrical impedance device, which were recorded by the operator. The radiographic image and raters’ calibration processes were done on ten initial cases with the help of a dental radiologist. The measurement was executed by drawing a straight line from the deepest point of the carious tissue floor to the highest point of the pulp before and after ending of the tooth cavity preparation [17] Fig. 2.

### Patient-reported outcome measures (PROMs): patients satisfaction

Patients were evaluated regarding their satisfaction with the handling and conveniency of each device using a 4-point Likert scale: (a) “yes, very satisfied”, (b) “yes, mostly satisfied”, (c) “less satisfied”, (d) “not at all satisfied”. They were also asked to mark the Visual Analog Scale (VAS), which is a 100 mm straight horizontal line with the left end representing “not at all satisfied” while the right end indicating “very satisfied”. The satisfaction value was calculated by measuring the distance from the left end of the scale to the mark in millimeters and expressing it as a percentage (10 mm equals 10%, 20 mm equals 20%, etc.) [18].

### Data treatment and statistical analysis

In order to obtain common units for comparison and based upon a previous study [19], electrical impedance device “Prepometer™” green LEDs were representing 2.1 to 3.0 mm radiographic RDT, yellow LEDs to 1.5 to 2.1 mm, orange LEDs to 0.9 to 1.5 mm while red LEDs to less than 0.9 mm. Based on these measurements, cavities depth was divided into shallow preparations where radiographic RDT is greater than 2 mm, moderately deep preparations when the RDT is 1–2 mm, deep preparations when the RDT is less than 1 mm while very deep preparations when the RDT is less than or equal to 0.5 mm [20].

Categorical and ordinal data were presented as frequency and percentage values. Numerical data were tested for normality by checking distribution and by using Shapiro–Wilk's test. Normally distributed data were presented as mean and standard deviation (SD) values, while non-parametric data were presented as median and interquartile range (IQR) values. Inter and intragroup comparisons were analyzed using signed rank test, while agreement between devices and observations was tested using weight kappa (WK) coefficient. Statistical analysis was performed with R statistical analysis software version 4.3.1 for Windows (R Core Team, 2023).

## Results

The study was conducted on 70 cases (i.e., 33 males and 37 females) with the mean age of (18.71 ± 4.79) years. A summary of demographic data is presented in Table 1. Radiographic data were measured twice and there was a strong statistically significant agreement between both observations (WK = 0.955 (95% CI; 0.905–1), *p* < 0.001).

**Table 1:** Demographic data

*Parameter*		*Value*
***Sex [n (%)]***	***Male***	33 (47.14%)
	***Female***	37 (52.86%)
***Age (Mean* ± *SD) (years)***		18.71 ± 4.79
***Treated arch [n (%)]***	***Upper***	29 (41.43%)
	***Lower***	41 (58.57%)
***Treated tooth [n (%)]***	***Premolar***	15 (21.43%)
	***Molar***	55 (78.57%)

A cumulative Link Mixed Model (CLMM) was built to analyze the interaction of different tested variables (i.e., predictors) with the remaining dentine bridge thickness (i.e., outcome). The random intercepts for individuals had a variance of 2.76 and a standard deviation of 1.66, highlighting the importance of the random variable in accounting for unobserved heterogeneity within the model. Results showed that using the Prepometer™, measuring after excavation, and having an older age were all significantly associated with an increased cavity depth (i.e., thinner dentine bridges) (*p* < 0.05). In addition, they showed that gender and type of treated tooth had no significant effect on dentine bridge thickness (*p* > 0.05) Table 2.

**Table 2:** A Cumulative Link Mixed Model (CLMM) to analyze the interaction of different tested predictors with the remaining dentine bridge thickness outcome

*Variable*	*Coefficient*	*95% CI*		*SE*	*z-value*	*p-value*
		**Lower**	**Upper**			
***Age***	0.09	0.01	0.18	0.04	**2.14**	0.032*
***Sex (female)***	0.00	-0.97	0.97	0.49	**0.00**	**0.999**
***Treated tooth (molar)***	0.54	-0.83	1.90	0.70	**0.77**	**0.439**

^*^Significant (*p* < 0.05), *CI* Confidence interval

**Fig. 3:**
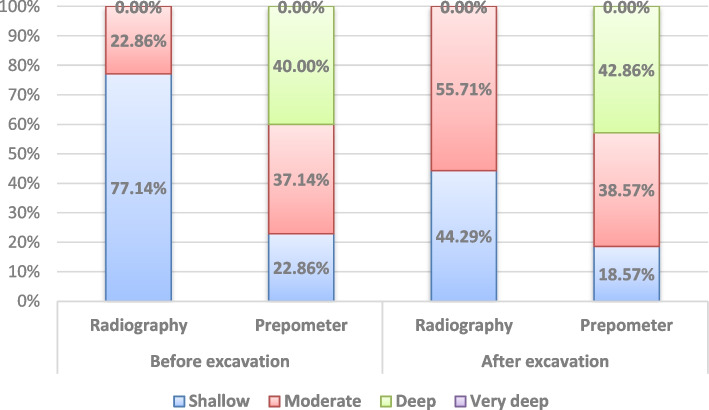
Stacked bar chart showing readings of both techniques

Results of readings made by both techniques are presented in Table 3 and in Fig. 3. Before excavation, most of the cases were judged radiographically to have cavities with shallow depth, whereas after excavation there was significant increase of cases diagnosed with medium depth cavities (*p* < 0.001). However, for the Prepometer™, before and after excavation, most of the cases were judged to have deep cavities and the difference was not statistically significant (*p* = 0.461).

**Table 3:** Inter, intragroup comparisons and agreement of remining dentine bridge thickness measurements

*Time*	*Remaining dentine bridge*	*n (%)*		*Test statistic*	*p-value*	*Weighted kappa (95% CI)*
		***Radiography***	***Prepometer™***			
***Before excavation***	***Shallow***	54 (77.14%)	16 (22.86%)	**49.50**	< 0.001*	**0.018 (-0.059:0.095)**
	***Moderate***	16 (22.86%)	26 (37.14%)			
	***Deep***	0 (0.00%)	28 (40.00%)			
	***Very deep***	0 (0.00%)	0 (0.00%)			
***After excavation***	***Shallow***	31 (44.29%)	13 (18.57%)	**22.50**	< 0.001*	0.200* (0.097:0.302)
	***Moderate***	39 (55.71%)	27 (38.57%)			
	***Deep***	0 (0.00%)	30 (42.86%)			
	***Very deep***	0 (0.00%)	0 (0.00%)			
***Test statisti*c**		**28.00**	**185.00**			
***p-value***		< 0.001*	**0.461**			

^*^Significant (p < 0.05), *CI* Confidence interval

Meanwhile, the results of intergroup comparisons showed that, before and after excavation, there was a significant difference between measurements made by both techniques (*p* < 0.001). After excavation, there was a weak agreement between measurements (WK = 0.2, *p* < 0.001), while before excavation, the agreement was not statistically significant (*p* = 0.407).

**Fig. 4:**
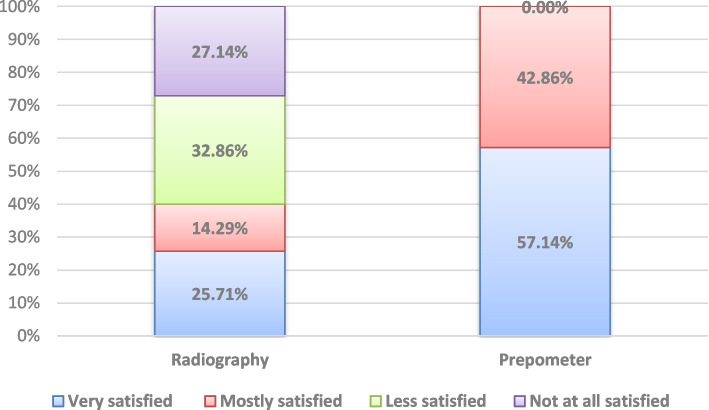
Stacked bar chart showing patient satisfaction toward both devices handling

**Fig. 5:**
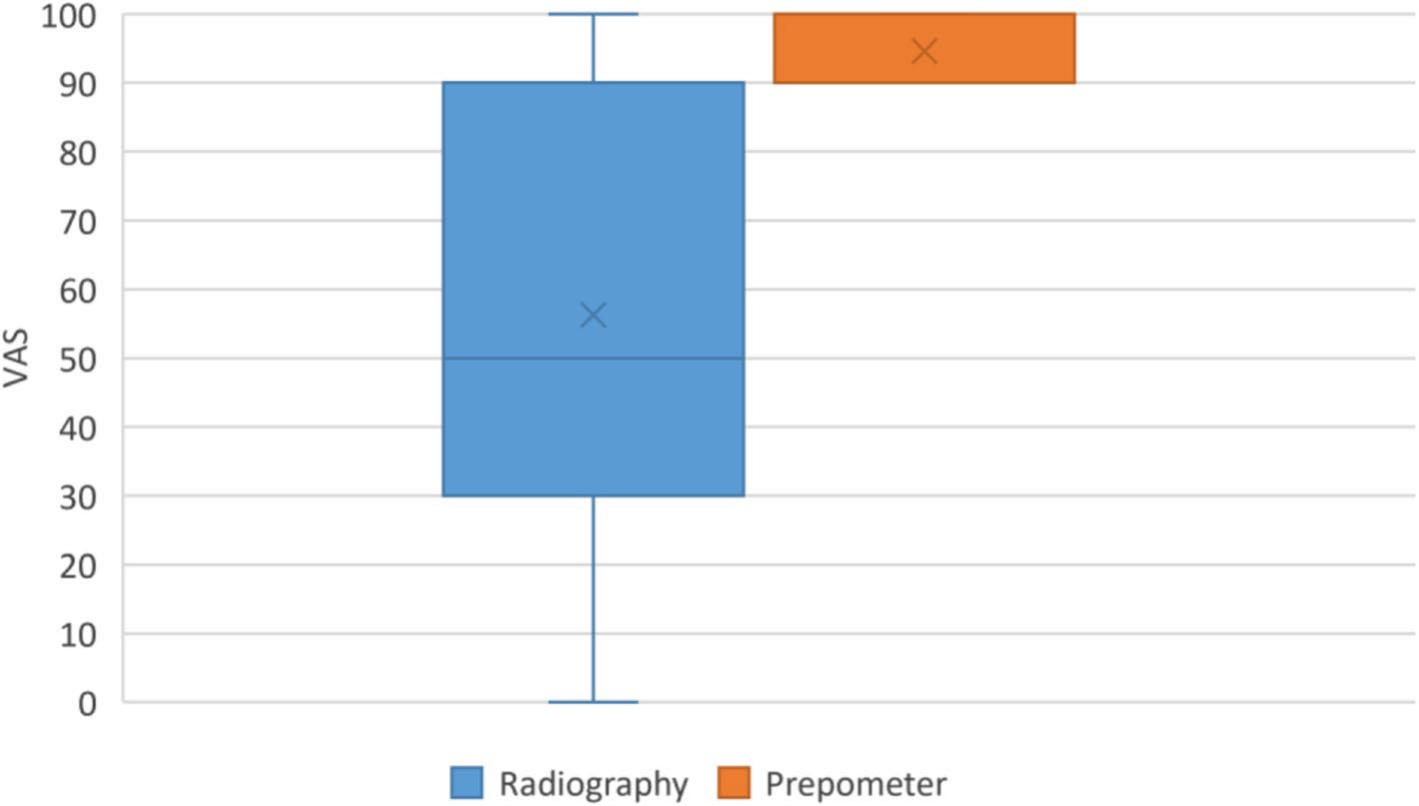
Box plot showing VAS values

Results of intergroup comparisons for patient satisfaction and VAS presented in Figs. 4 and 5 respectively showed patients to have significantly higher satisfaction with the Prepometer™ (*p* < 0.001).

## Discussion

Estimation of the RDT is crucial during tooth preparation to guard the pulp vitality and determine the most appropriate restorative options. As the most commonly used tool for measuring RDT by dental practitioners is radiography [21], digital radiography was used in this study as a reference test. Digital radiography offers sharper, clearer adjustable images than the conventional method due to the use of sensitive plates. Additionally, it allows for digital line measurements by the associated software [22]. The periapical radiograph type was chosen as it is frequently used in routine dental treatment and provides detailed images for the related periapical region meanwhile, a standardized parallelling device was utilized to allow for more accurate measurement of the vertical and horizontal directions [23]. Adjustment of image brightness and contrast was performed to enhance the characteristic carious and cavity preparation boundaries, and this was supported by studies [24, 25] that found that digital image enhancement increased the radiographic diagnostic accuracy. Calibration of the raters was done with the help of an experienced radiologist as recommended by Schwendicke et al., [26] who reported that visual detecting of deep carious lesions on radiographs dictating a certain level of expertise due to wide variations and subjectivity in examiners’ performance.

The electrical resistance based device “Prepometer™” was found to be an accurate measuring tool for estimation of RDT in comparison to histological sectioning and CBCT methods [12]. In order to decrease the confounders that could affect its accuracy, young adult and early adult age groups were selected as representative for conditions with normally wide dentinal tubules, less sclerosis and high dentinal fluid [27] where electrical impedance would be expected to be low [5, 10]. Meanwhile, the interaction between different tested predictors in this trial on the remaining dentine bridge thickness outcome showed that older age patients within the selected age range were all significantly associated with an increased cavity depth (i.e., thinner RDT), this could be attributed to tendency of older patients to neglect early treatment of their carious teeth until they become deeper and symptomatic due to their heavy workload. Gender and type of treated tooth had no significant effect on dentine bridge thickness. This was in accordance with Gasqui et al., [17]; there was no association between type of tooth and radiographic measurement of RDT also with Al Jhany et al., [28] who reported absence of significant differences between premolars and molars as both have similar RDT ranges. All teeth cavity preparation was performed using a coarse grit carbide bur to decrease the influence of the smear layer which could be entrapped inside the dentinal tubules, as explained by Violich et al., [10] who found that the particle size of the smear layer influenced the accuracy of the Prepometer™ measurements.

Regarding the results of this diagnostic accuracy study comparing the digital radiography and the electrical impedance-based device “Prepometer™” for estimation of RDT, the intragroup comparison showed that cavity depth of most of the cases was underestimated radiographically indicating overestimation of the RDT. This could be attributed to the inherent weakness of periapical radiography being a 2D image for a 3D object and the possible superimposition of dentinal areas of different mineralization levels against the direction of the X-ray beams. This was in line with the findings of Lancaster et al., [6], Kooistra et al., [29] and Khalaf et al., [30]. The later found that even the digital bitewing radiograph underestimated the true clinical depth of proximal carious lesions. Conversely, Berbari et al., [4] reported underestimation of RDT radiographically. However, the recent integration of a type of artificial intelligence model, the convolutional neural network (CNN), into digital dental radiography has shown outstanding performance in computer vision and become widely used for assessing visual imagery [31].

Still, for the Prepometer™, pre-operative and after excavation, there were consistent results, and the difference was not statistically significant (*p* = 0.461). This was in accordance with Purton et al., [5] who reported in their laboratory study that it was a reliable predictor of pulp approximation also with Sarhan et al., [12] who supported the accuracy of the device in estimation of RDT in relation to the true histologic depth. In contrast, Tielemans et al., [13] reported in their in vivo-invitro pilot study that although the device was reproducible, but it was not correlated with the RDT. Still, this conclusion could not be generally ascertained due to the small sample size of the study as only two old patients with twelve teeth were analyzed. These results of Prepometer™ may spot the light on its potential role in helping dentists especially those inexperienced to easily speculate the cavity depth from the beginning also the caries excavation endpoint.

Regarding the intergroup comparisons, the agreement was not statistically significant different between preoperative measurements taken by the two techniques, while poor agreement was observed after excavation where medium and deep cavities were dominant. Therefore, the Prepometer™ could be of benefit in determining the deep caries excavation endpoint. This difference could be due to the inherent limitations of radiography as aforementioned. In addition to, the lack of commonly accepted distinct tactile or radiographic millimeter scale for categorizing carious lesion depth (e.g. shallow, medium vs deep). This difference in depth thresholds between the two modalities could affect the level of agreement.

The patient satisfaction results showed that the patients were significantly satisfied with the Prepometer™; this could be attributed to its reduced procedural time, lower cost, being noninvasive, no radiation exposure, and no need for insertion of any tools that may distress the patient or stimulate gagging reflex. These advantages combined with the positive patient experience may warrant the potential role of this device in increasing the level of patient awareness of the iceberg nature of dental caries and how early intervention could save a lot.

Based on the current results, the proposed hypothesis is rejected. Limitations of the study include primarily lack of the true gold standard method for RDT determination, which is the histological validation, relatively the small sample size, including only posterior teeth and the inevitable human errors due to the absence of standard tooth cavity measurement point. Thus, further clinical research is recommended with larger sample size, on adult and geriatric age groups where dentin sclerosis may affect the Prepometer™ electric current. In addition, long follow up periods for restorations placed according to the Prepometer™ based treatment decisions are needed.

## Conclusion

Under the limitations of the current trial, it could be concluded that.

1. The electrical impedance-based device “Prepometer™” could be a viable option for clinically determining RDT with high patient satisfaction.
2. Digital periapical radiography tended to overestimate the RDT in relation to the electrical impedance-based device “Prepometer™”.

## Data Availability

The datasets used and/or analyzed during the current study are available from the corresponding author on reasonable request.

## Abbreviations

RDT: Remaining Dentin Thickness
OCT: Optical Coherence Tomography
CBCT: Cone Beam Computed Tomography
ICDAS: International Caries Detection and Assessment System
ICCMS: International Caries Classification and Management System
PSP: Photostimulable phosphor plate
PROMs: Patient-reported outcome measures
CNN: Convolutional Neural Network
CLMM: Cumulative Link Mixed Model
